# Supporting Patients with Psychosis in the Community To Stand Up and Move More: Perspectives of Community Mental Health Staff

**DOI:** 10.1007/s10597-025-01563-9

**Published:** 2025-12-27

**Authors:** Rowan Diamond, Felicity Waite, Anne-Marie Boylan, Alice Hicks, Thomas Kabir, Daniel Freeman

**Affiliations:** 1 Oxford Cognitive Approaches to Psychosis (O-CAP), Department of Experimental Psychology, University of Oxford, Life and Mind Building, Oxford, OX1 3EL UK; 2 Oxford Health NHS Foundation Trust, Oxford, UK; 3 Nuffield Department of Primary Care Health Sciences, University of Oxford, Oxford, UK; 4 The McPin Foundation, London, UK

**Keywords:** Clinicians, Physical activity, Schizophrenia, Sedentary

## Abstract

People with psychosis typically show high levels of sedentary behaviour and low levels of physical activity. Effective interventions are needed, and staff will play a crucial role in implementation. Aims: To understand staff perspectives on reducing sedentary behaviour and increasing physical activity. Eighteen staff from NHS mental health trust community teams were interviewed, with data analysed using reflexive thematic analysis. Four themes were developed: (1) Choosing to target movement: staff recognise the need to address movement but struggle to prioritise it; (2) Encouraging but not steamrolling: balancing encouragement without pushing too hard is essential for motivation; (3) Tapping the reservoir of staff knowledge: staff possess valuable expertise to leverage; (4) Using lived experience: lived experience accounts effectively motivate and inspire hope. Despite recognising the importance of the issue, limited resources in services hinders prioritisation of increasing patient movement, and interventions are often not attempted. Adapting routine practices and recruiting support (e.g. from willing carers) may increase intervention success without burdening staff.

## Introduction

Patients diagnosed with psychosis are typically more physically inactive and spend more time sitting and less time exercising than other people in the general population (Stubbs et al., 2016; Stubbs, Williams, Gaughran, & CraiStubbs et al., 2016a, b, c). This can contribute to poorer physical and mental health (De Hert et al., 2009; Hjorthoj et al., 2017; Rosenbaum et al., 2014). When physical activity is increased for people with psychosis there have been multiple benefits shown (Firth et al., 2015) meaning that increasing physical activity is a target in UK national health service guidance (NICE, 2014) and strategic planning (NHS England, 2024). Increasing physical activity in patients with psychosis has continued to be an important focus of research as part of an attempt to close the gap in physical health and mortality figures. However, efforts to increase exercise have often struggled to engage patients, and have experienced high levels of drop-out (Firth et al., 2015; Romain et al., 2020). Further developing our understanding of the causes of inactivity (e.g. what leads to and maintains sedentary behaviour) may lead to improvements. In particular, we aimed to gain the perspective of staff, who will be responsible for delivering or supporting real world interventions.

Social support is a particularly important factor in initiating and maintaining physical activity for people with psychosis (Carney et al., 2017; Firth et al., 2016; Soundy et al., 2014; Ussher et al., 2007). The support received from healthcare staff not only includes valuable social support, but also includes interventions to support patients to reach their goals, which may include being more physically active. Research has shown that exercise interventions delivered by specialist exercise professionals have better adherence and success than those delivered by non-specialists (Firth et al., 2015; Lederman et al., 2020). This type of specialist support is often unavailable in community mental health teams (CMHTs) (Vancampfort et al., 2017), leading to calls for more exercise professionals to be integrated into mental health teams and to increase the competencies of staff at all levels (Firth et al., 2019; Vancampfort et al., 2017).

At present in the UK, CMHT staff are likely to have the majority of conversations with community patients about safeguarding their physical as well as mental health, including discussions about their physical activity and sedentary behaviour. In previous qualitative studies, factors identified as helpful for increasing activity for people with psychosis include staff knowledge, staff modelling, staff prioritising patients’ physical health, creating good relationships, and the provision of daily routine (Leutwyler et al., 2013; Mucheru et al., 2019). Studies seeking the perspective of staff focus on exercise, but there has been less discussion about attempts to encourage patients to stand up more often in order to break long periods of sedentary time. Given the independent links of exercise and sedentary behaviour with physical health outcomes (Biswas et al., 2015), it will be important to pursue staff perspectives on both.

In a qualitative study of patients’, carers’, and staff perspectives, a framework for increasing exercise and decreasing sedentary behaviour in people with psychosis was developed (Diamond et al., 2024). Five factors were found to promote standing and physical activity: Purpose, Predictions, Present state, Provision, and Process. In this paper we report an analysis specifically of the staff perspective from the interviews. Our aim was to understand the views of community mental health staff on addressing sedentary behaviour and supporting physical activity for people diagnosed with psychosis.

## Materials and Methods

### Study Design

This qualitative study used reflexive thematic analysis (RTA), (Braun & Clarke, 2022), to analyse the data. RTA is a qualitative research method used to identify and interpret patterns of meaning across a dataset. It was chosen as it can accommodate combining peer and non-peer researcher perspectives, and combining data from focus groups and interviews. The study was designed and analysed taking a critical realist approach. This approach postulates that whilst there is an objective reality, this is observed through the researcher’s unique lens.

The study was conceptualised, designed, conducted, and analysed in collaboration with people with lived experience, including an expert advisory group and a peer researcher who co-facilitated focus groups. Criteria for demonstrating credibility in qualitative research (Yardley, 2008) informed the study throughout.

Staff participants took part alongside patients diagnosed with psychosis and carers. This paper specifically focusses on data provided by staff. The perspectives of the other groups are presented elsewhere (Diamond et al., 2024; Diamond, Waite, Boylan, Hicks, Kabir et al., 2024).

The study was approved by the Health Research Authority Wales Research Ethics Committee 6 (REC reference: 21/WA/0285). All participants gave their informed consent prior to their inclusion in the study.

### Participants

Participants were recruited via three mental health trusts in England (Oxford Health NHS Foundation Trust, Berkshire Healthcare NHS Foundation Trust, and Humber Teaching NHS Foundation Trust). They were identified by clinical research network (CRN) staff or identified themselves.

Inclusion criteria were: aged 16 years or older, able to give informed consent, experience of working in the community in the past year with patients who had a clinical diagnosis of schizophrenia spectrum psychosis and experience of psychosis in the past two years.

Purposive sampling was used to maximise variation in participant characteristics, including job role and type of community mental health team (e.g. Early Intervention Team or CMHT).

### Procedure

Participants could choose to take part in an individual interview or focus group with patients and carers.

After sharing their backgrounds and interests in the research area, online and in person focus groups and interviews were conducted by a clinical psychologist (RD) and co-facilitated by a peer researcher (AH), both trained in qualitative research.

The topic guide (see supplementary materials), developed in consultation with the PPI Expert Advisory Group, covered sitting, standing up to break sitting time, exercising, and how people can be encouraged to move more. It was employed flexibly, to allow for the development of new ideas during participation. Follow up questions and prompts were used as needed.

Field notes and audio-recordings were taken, transcribed verbatim, and pseudonymised.

### Analysis

Analysis, with a critical realist perspective, was informed by the six-step guidance provided by Braun & Clarke (Braun & Clarke, 2022).

Following analysis of the total dataset (patients, carers, and staff), the codes pertaining to staff issues were separated. Coded extracts and then each relevant transcript were reread in detail, focussing specifically on the issues of supporting movement and physical activity. This process resulted in the addition of new codes. The list of all codes was then discussed with members of the research team. New thematic clusters, based solely on staff issues, were generated, discussed and re-generated iteratively, through a process of reflection and immersion in the data.

Analysis was supported by use of NVivo 12 (QSR limited, 2018).

## Results

Between 17th December 2021 and 1 st June 2022, eighteen staff members (Table 1) participated.

**Table 1:** Participant characteristics

	*n*	%
**Age range**		
16–25	1	6
26–35	6	33
36–45	7	39
46–55	2	11
56–65	1	6
66+	1	6
**Gender**		
Male	4	22
Female	14	78
**Ethnicity**		
White British	15	83
White other	1	6
Black African	0	0
Pakistani	0	0
Bangladeshi	1	6
Other Asian	1	6
Other mixed background	0	0
**Team type**		
Early Intervention Service	11	61
Adult Community Mental Health Team	7	39
**Role**		
Care Co-ordinator: Occupational Therapist	2	11
Care Co-ordinator: Nurse	3	17
Support worker	4	22
Psychiatrist	2	11
Psychologist	5	28
Physical health support worker	2	11

Of the eighteen staff, nine took part in focus groups. Apart from one group, these consisted of staff, patients, and carers together, allowing interactions in the group to contribute to learning. With the exception of one pair (in which a staff member participated with a patient they worked with), participants did not know one another. Facilitators did not know the participants.

Four overarching themes were developed. The first, “choosing to target movement” is about the need for staff to attend to patients’ movement, and the challenges that this entails. The second, “encouraging but not steamrolling” describes the choices staff face about how to gauge their efforts to reduce sedentary behaviour and increase physical activity. The third, “tapping the reservoir of staff knowledge” describes the techniques and knowledge staff need to support movement. The fourth theme, “using lived experience” highlights the value of lived experience in helping patients to feel hopeful and understood.

### Choosing To Target Movement

Staff members described experiencing competing demands for their time when working with patients, and often feeling that their limited resources had to be directed towards risk management and management of acute mental health issues (in which positive symptoms of psychosis were prioritised), as opposed to supporting physical health and movement. Staff often felt that they simply did not have the time for supporting movement, despite identifying it as an important need. To help with this tension staff said that they felt they needed to gain permission to integrate physical activity into their work, for example, from managers, and there was a degree of feeling that focussing on physical activity was “not my role”. In the context of limited staff resources (staff, facilities, funding, time) and patient resources (e.g. money, transport, support), prioritising movement and physical activity became more challenging and occasionally meant that opportunities were missed, which ultimately led to lost momentum.

*Again just having more time because I think there’s that period of time when someone’s got the motivation and the energy and things and then if you’re not able to harness it then and there it feels like it kind of just slips away and then it’s gone and you’re like okay I’m going to have to start from scratch again now – Jen*.

*We’re too busy… we’re just short staffed at the moment and it’s difficult to hire people so our caseloads are just getting bigger and what we’re expected to do is getting more and more and you basically do have to reign it in to the real essentials – Jen*.

*I think there is competing demand so*,* you know*,* I don’t have that much time to focus on physical health ‘cos I’m always seeing people who are unwell and I need to make sure I’m doing a really good*,* safe assessment …so I don’t get the time ….I know it’s very important…– David*.

Staff also acknowledged that discussions, even in passing, about the impact of prolonged sitting very rarely took place, either with patients or between staff.

Staff felt that patients also needed to prioritise physical activity, alongside other areas in their life. Staff said that sometimes patients could be so focussed on recovery from their illness that they put their lives “on hold” and stopped doing any of the things they would usually like to be doing.

*[Patients’] focus is really on the psychosis*,* kind of*,* “once that’s better*,* I can get back to my life” and that involves exercise*,* that involves doing other things to look after myself but for now*,* it’s the psychosis. I need to find a way to help me recover from it – Mariam*.

### Encouraging but not Steamrolling

Staff described a tension between creating motivation through encouraging activity, and putting too much pressure onto patients, thus demotivating them. They described a thoughtful process of deciding how to calibrate their support; not doing too much to support movement (for example pushing too hard, coercing people to move, or fostering a dependence) but doing enough to overcome some of the barriers to movement. This balance felt difficult to achieve as each person has unique needs, which vary over time; an attempt to encourage activity might go too far, and risk the patient resisting and doing less than they would have done prior to the intervention, whereas on another occasion, this same intervention might be the trigger to get someone started on moving in a pleasurable way.

*You want to encourage but you also don’t want to give up too easily. It’s a real balance*,* isn’t it? Not expecting too much from a person*,* but getting them that push when you know it’s the right time to. It’s a difficult one*,* that one – Cathy*.

*I don’t think being too directive really helps*,* because people just feel a bit steamrolled and get a bit resistant and dig their heels in – Jen*.

*When somebody’s very flat and really struggling*,* if we didn’t keep nudging*,* it just won’t happen spontaneously*,* you know*,* it just doesn’t happen and there’s something about …… It is a fine line ….but I just think people do need to be nudged a little bit and saying no is often a symptom of a condition rather than actually a personal choice… they’re so flat it’s just easiest to say no and be left alone but it just won’t change without a wee bit of encouragement – Maggie*.

*If You’re Going Too hard, You’re Going To Make Them Feel little – Mae*

### Tapping the Reservoir of Staff Knowledge

Staff were able to identify many things that could be done to facilitate movement, despite the confines of a stretched NHS. Opportunities to incorporate conversations about standing up and physical activity were discussed including asking about it routinely in physical health checks. However, this would involve training all staff, including educating them about the impact of sedentary behaviour.

*I think potentially you’d like more awareness and training and then healthcare professionals could be helpful – Maria*.

Routine care could also be adapted so that it included activity as part of normal interactions, e.g. appointments that involved walking and talking outside. Whilst some staff might already do this, others may need training in how to manage this, including reassurance that this was acceptable to management.

*If my manager thought it was okay for me to go on a park walk with my patients this afternoon*,* I would absolutely – Izzy*.

Staff also described the effect of valuing movement themselves. They recognised that promoting movement in others is influenced by one’s own views of it, and these could be influenced in turn by their own behaviour change.

*Other people would be quite quick to say*,* “well I don’t do any exercise” and it becomes much harder to enthuse about it I think. I think like as a team*,* we would do better if we were more active just generally and promoted that – Sally*.

Other ways of motivating patients which staff could learn and develop were the use of careful language and thinking about the things that matter to that person. Sometimes a patient’s physical health was not their priority, and explaining the value of movement for mental health may be a better motivator. Furthermore, recognising success and having good ways of measuring this was also valuable. Good support was gentle yet firm and persistent in the face of setbacks.

The importance of psychological principles and techniques was emphasised, including the value of psychoeducation, identifying the right motivators for patients (and employing motivational interviewing techniques to establish these), identifying and working with thoughts and identifying vicious cycles.

*I suppose helping people to make those links where they are doing exercise*,* however small it is*,* making it a bit more explicit with them so kind of reminding them in sessions*,* just really drawing out those little cycles where people are saying they’re doing exercise*,* they’re not getting these thoughts and it’s helping them to continue exercise*,* it’s helping them to feel better – Mariam*.

*I think we’re trying to think a bit more psychologically about it because they are being met with quite a lot of resistance and people disengaging because the work is too hard*,* so we’re trying to look at that more psychologically and think about why these people are struggling so much with the physical activity because it’s not just a case of ‘right*,* let’s get up and moving’ because there might be a lot more to it … It is looking more at thoughts and feelings and how they’re interacting with each other and then looking at the action after that because the action is obviously the thing that we want to improve*,* to get them up and about but we know that the thoughts and feelings*,* like the ones that I’ve explained already regarding feeling at risk and feeling like someone’s going to physically hurt them*,* we’ve got to deal with that first before we try and get them up – Simon*.

Goal-setting and careful grading of the steps towards physical activity were commonly used, all contributing to a tailored approach to increasing physical activity. If already in place, these skills are invaluable. However, many staff may not have these skills unless they are taught them.

Where specific job roles exist for which supporting physical activity is a key part, this can be effective. This may be because they possess specialist skills or because they are able to prioritise movement as a treatment target.

*What I’ve noticed is having people whose specific job it is to do it seems to work very well – David*.

Staff also recognised that patients’ family members could be effective at engaging and motivating them, and working together with them was hugely important. This included informing friends or family of the intervention so that they can maintain the work outside of the staff-patient interaction.

*What’s really helpful for people is having the professionals*,* their friends and family on the same page…. kind of singing from the same hymn sheet. A lot of the work that we do with patients*,* we often include their friends or family or people that they live with*,* in that work for them to keep it going when we’re not there. That can be really helpful*,* because sometimes it takes someone’s friends or family to make that change in them*,* rather than a professional…. they love them*,* they care about them more than they would like me*,* a random person going in – Rosie*.

### Using Lived Experience

Staff recognised the significant challenges involved in getting more active, specifically for people with psychosis, and that these challenges may not be fully understood by people who don’t have personal experience themselves. The value of receiving accounts from people who have managed to overcome these challenges was noted, along with the powerful hope that these accounts can engender.

*It gives people a bit of hope as well*,* and it makes them think ‘maybe I can get better*,* maybe I can do more’. It’s that hope for the future*,* which is massively important in motivation to do anything*,* really…. We do a lot of work with patients*,* showing them there is a light at the end of the tunnel. We have groups and things that people can attend*,* with people who have been through what that person had been through*,* lived experience. I know patients find that quite beneficial*,* just hearing that ‘they were where I was and now look at them*,* actually maybe I can get there as well’. Just sharing stories with people can be really helpful. We’ve all been moved by what [patient participant]’s been saying today*,* and if service users could hear that*,* they would find that really helpful and go ‘look how far he’s come*,* I want to be like that’– Rosie*.

*Give them hope isn’t it*,* so you know that it’s doable*,* it’s accessible*,* that somebody that’s had similar experiences to them has managed to come through the other end*,* you know*,* it’s a different….it’s a different*,* um*,* conversation when you’re speaking to somebody that’s experienced something that you’ve experienced because you know they’re coming from a place of knowledge…. that it’s not as easy as people think of just getting up and doing it… I think the peers thing is a great idea*,* I…I …I think we don’t have enough of that – Maggie*.

## Discussion

Community mental health staff described many ways they could influence the sedentary behaviour and physical activity of patients, although in order to do so, they perceived the need to have permission from their service to prioritise this, skills to offer the right amount of encouragement, and other skills and resources to deliver the interventions. Whilst the physical activity of patients in community teams has previously been found to be deprioritised in favour of other duties (Happell et al., 2012; Leutwyler et al., 2013; Soundy et al., 2014; Vancampfort et al., 2016) it has also been argued that it is a duty of care to safeguard the physical health of patients (Firth et al., 2019). But where community teams often don’t have access to specialist physical activity practitioners, and have a shortage of resources to even do the “bare minimum” of care for their patients’ mental health, this remains a considerable challenge. Any change in demands or prioritisation in care raises ethical and strategic questions such as how to reallocate resources whilst balancing long and short-term benefits to patients, and consideration of both their mental and physical health. Suggestions raised by staff in this study, for example, staff informing themselves about the effects of sedentary behaviour, and changing their own attitude towards it, training up the workforce so that everyone is aware of the effects of sedentary behaviour, and learning ways to help intervene e.g. psychological techniques to help patients make progress, will need to be carefully considered by services.

Consistent with previous findings (Glowacki et al., 2019), our results suggest that community teams supporting increases in patients’ physical activity could require systemic change. Change in staff knowledge and beliefs about the benefits of physical activity are important in order for it to be recommended to patients. This may be particularly the case when it comes to knowledge about the impact of sedentary behaviour, as our findings suggested that this is rarely discussed between staff or with patients. Service level change may be necessary to ensure that the impact of sedentary behaviour is understood and considered throughout service delivery.

Staff training, which has also been called for by many others (Carlbo et al., 2018; Leutwyler et al., 2013), could highlight the impact of sedentary behaviour. Staff training could also include developing skills in approaching an intervention at the right level for the patient – not so much encouragement that the attempt becomes counter-productive, but also enough that people are supported to make progress.

Staff beliefs and attitudes are likely to be influenced by their own experiences, and our findings suggest that, as other studies have found (Leutwyler et al., 2013), the modelling behaviour of staff is important in effecting change for their patients. Staff who are interested and enthusiastic about movement are likely to be more effective at encouraging it in patients (Vancampfort et al., 2013), and health care practitioners who have healthy lifestyles are more likely to recommend such behaviours to patients (Kleemann et al., 2020; Lobelo & de Quevedo, 2016). It has been suggested that enhancing staff training programmes to include a specific focus on staff health could enhance their efficacy (Rosenbaum et al., 2020). Similarly, the lived experience of people who have overcome barriers to reduce sedentary behaviour and move more provides encouragement and hope and is identified as an important component in interventions that could be used more. The impact of peer support interventions to improve physical health is equivocal (Coles et al., 2022; Stubbs et al., 2016c) and is an area for further research.

In addition to modelling physically active behaviours, staff highlighted that they can also influence patient behaviours by making movement a routine expectation as part of community team engagement (e.g. having appointments whilst walking). This small systemic change, alongside staff modelling, relates to the importance of patients’ expectations, and how these are shaped by other people in their environment (Diamond et al., 2024).

The encouragement of more frequent standing (as opposed to encouraging exercise) is perhaps less time consuming, less specialist, and more consistent with other work carried out by community staff. A staff focus on more frequent standing time and substituting some sitting time with light physical activity may be less overwhelming for patients than aiming to achieve the recommended amount of exercise in a week. This focus would be consistent with the message that “something is better than nothing” (Powell et al., 2011). This perspective naturally places the expectation of physical activity somewhere in the middle of a continuum, which may aid in combatting the black and white thinking that can prevent patients from doing any physical activity at all.

Staff frequently described the importance of psychological thinking in supporting patients to stand more often and to move more. They recognised that whilst there could sometimes be excellent opportunities for movement in the community, often these are inaccessible to someone due to psychological barriers such as anxiety, low motivation or low mood. Indeed, this was demonstrated in a pilot intervention in which patients were given free access to a gym, a physical activity trainer, bus and parking tickets, and yet by the end of the study period, 90% of participants had dropped out (Archie et al., 2003). The main reason given for the drop out was low motivation. In the present study psychologically trained and non-trained staff alike described ways in which they thought psychological understanding and techniques could be used to good effect. The application of psychological techniques to increase physical activity is described elsewhere (Diamond & Waite, 2021). Cognitive Behavioural Therapy (CBT) has previously been suggested as an important tool in increasing exercise self-efficacy, especially amongst outpatients (Ussher et al., 2007). Staff training, incorporating some basic psychological techniques could prove a valuable addition to routine care.

Although psychological understanding and techniques are highlighted by these findings, methods for quickly assessing problematic patient beliefs about physical activity are only just emerging (Diamond et al., 2025). Future research is needed to establish the impact of identifying and targeting beliefs that inhibit movement amongst patients with psychosis.

Based on our findings and participant suggestions, Table 2 contains a number of suggestions for supporting patients.

**Table 2:** Suggestions for supporting patients

	Possible approaches
Choosing to target movement	• Increase service level awareness of the importance of sedentary behaviour • Routine staff training about the benefits of breaking up sedentary time, as well as of exercise • Routine questioning about activity in health checks • Active appointments where appropriate and managerial support for this • Provision of information to patients and carers about the importance of movement
Encouraging but not steamrolling	• Training for staff to learn techniques (e.g. motivational interviewing*) to pitch the intervention at the right motivational level
Tapping the reservoir of staff knowledge	• Careful assessment of individual needs, preferences, and psychological barriers • Interventions tailored to meet these needs • Training in how to use some psychological techniques to help facilitate movement • Provision of physical activity interventions for staff (or joint interventions with patients) • Inclusion of new roles in workforce e.g. support workers focussing solely on physical health or activity • Close working with family/friends of patients - Sharing knowledge where appropriate - Provision of information about sedentary behaviour, physical activity and local resources
Using lived experience	• Provision of lived experience accounts (delivered in person or via other media) • Provision of peer support worker input into teams

*Motivational interviewing is an approach used to help patients to resolve their ambivalence about behaviour change. Evidence for its effectiveness in this context is limited, but evidence from other populations is promising

The findings of this study support the call for interventions to be tailored to fit the needs and preferences of the individual. Choosing the right level of intervention, the intervention techniques used and by whom are all done with the individual in mind. The number of considerations and decisions staff are making every day are illustrated by the detail of the responses they gave. These decisions are likely to be facilitated by close working with those who know the patient well (Diamond, Waite, Boylan, Hicks, Kabir, et al., 2024).

In a study examining carers’ perspectives (Diamond, Waite, Boylan, Hicks, Kabir, et al., 2024) the importance of close joint working between carers and staff is highlighted. This is also raised in the current study. Whilst for carers, their availability and keenness to do more to help is emphasised, for staff this is complicated. Whilst recognising the importance of this treatment target, they also described the limitations within which they work, particularly regarding time and resources. If it were possible to develop effective ways of staff and carers working closely together to increase physical activity in patients, this may be an effective way of reducing demand on staff time and allowing more targeted interventions, based on a detailed understanding of the patient and their life in the real world.

There are a number of study limitations. Participants were recruited from only three sites across the UK. As recruitment was via community mental health teams, the study did not cover work with patients managed in other care settings such as primary care or inpatient units. Although the sampling strategy was intended to maximise variation, the majority of participants were female and there was a higher representation of psychologists than other professional backgrounds. No qualified exercise professionals were available to participate, which is consistent with their representativeness in community teams. Participants all volunteered to participate and therefore may have had an interest in physical activity and promoting it. It is important to note that for these reasons they will not be representative of all staff working in the community. Staff participants in mixed focus groups may have contributed less to focus groups due to an awareness of the potential power imbalance. This may have resulted in them saying less or restricting their contributions. To overcome this, all participants were invited to get in touch afterwards if there was anything else they wished to contribute.
